# Correction: The Role of Diverse Strategies in Sustainable Knowledge Production

**DOI:** 10.1371/journal.pone.0168934

**Published:** 2016-12-16

**Authors:** Lingfei Wu, Jacopo A. Baggio, Marco A. Janssen

## Notice of Republication

This article was republished on December 7, 2016, to correct errors in the order of the figures. Please download this article again to view the correct version. The originally published, uncorrected article and the republished, corrected article are provided here for reference.

## Supporting Information

S1 File Originally published, uncorrected article. (PDF)

S2 File Republished, corrected article. (PDF)
