# Supplementary material for: The Role of Diverse Strategies in Sustainable Knowledge Production
Source: PLoS One. 2016 Mar 2;11(3):e0149151. doi: 10.1371/journal.pone.0149151 (PMC4775042; doi:10.1371/journal.pone.0149151)
Supplement: S1 File — (PDF) [file pone.0149151.s001.pdf]

Supporting Information

The TrueSkill algorithm

Invented as a chess rating system, Elo rating system [1] is widely used in games and team sports. Herbrich et al. developed the TrueSkill rating system by combining Elo's method with Bayesian inference and used it to match the competitors in Xbox online games (e.g., Halo 2) [2]. Liu Jing et al. applied the TrueSkill rating system to estimate the difficulties of questions as well as the skill levels of experts in online Q&A communities [3, 4]. They showed that this method overperforms other structure-based analysis such as the PageRank scores of users [5].

The TrueSkill system assumes that the "true skill' of each player satisfies a normal distribution of mean μ and variance σ, which are fixed to be the same for all players at the initiate step. Then the system updates the distributions of players according to the results of games. In particular, the system consider the current skills of two players (priori probability) and the winner-loser result (likelihood) to update the player skill levels (posterior probability). The equations used in the updating process are shown as follows (cited from [4]):

$$\mu_{winner} = \mu_{winner} + \frac{\sigma^2_{winner}}{c} v(\frac{t}{c}) \tag{1}$$

$$\mu_{loser} = \mu_{loser} - \frac{\sigma^2_{loser}}{c} v(\frac{t}{c}) \tag{2}$$

$$\sigma_{winner} = \sigma_{winner}[1 - \frac{\sigma^2_{winner}}{c^2} w(\frac{t}{c})] \tag{3}$$

$$\sigma_{loser} = \sigma_{loser}[1 - \frac{\sigma^2_{loser}}{c^2} w(\frac{t}{c})] \tag{4}$$

in which

$$t = \mu_{winner} - \mu_{loser} \tag{5}$$

$$c^2 = 2\beta^2 + \sigma^2_{winner} + \sigma^2_{loser} \tag{6}$$

$$v(t) = \frac{N(t)}{\Phi(t)} \tag{7}$$

$$w(t) = v(t)(v(t) + t) \tag{8}$$

N and Φ in Eq. 7 represent the PDF and CDF of a standard normal distribution, respectively. From the above equations we know that if the competition outcome is expected, i.e., the player of the higher skill level wins, the system will updates μ and σ slightly towards the positive direction (high score player obtains scores and low score player loses scores). On the contrary, if the outcome is unexpected, i.e. the player of the lower skill level wins, the system will update μ and σ dramatically towards the negative direction. The parameter β controls the updating effect; larger β leads to a more dramatic change. Figure S1 in S1 File visualizes the effects of updating rules on two example cases. The two plots in the upper row show how updates increase with surprise (smaller t) and the two plots in the lower row compare the resulting scores between expected (left) and unexpected (right) outcomes.

We apply the TrueSkill algorithm to the StackOverflow dataset, which contains over 18 millions of records (both of questions and answers) created in the period from July, 2008 to January, 2014. After

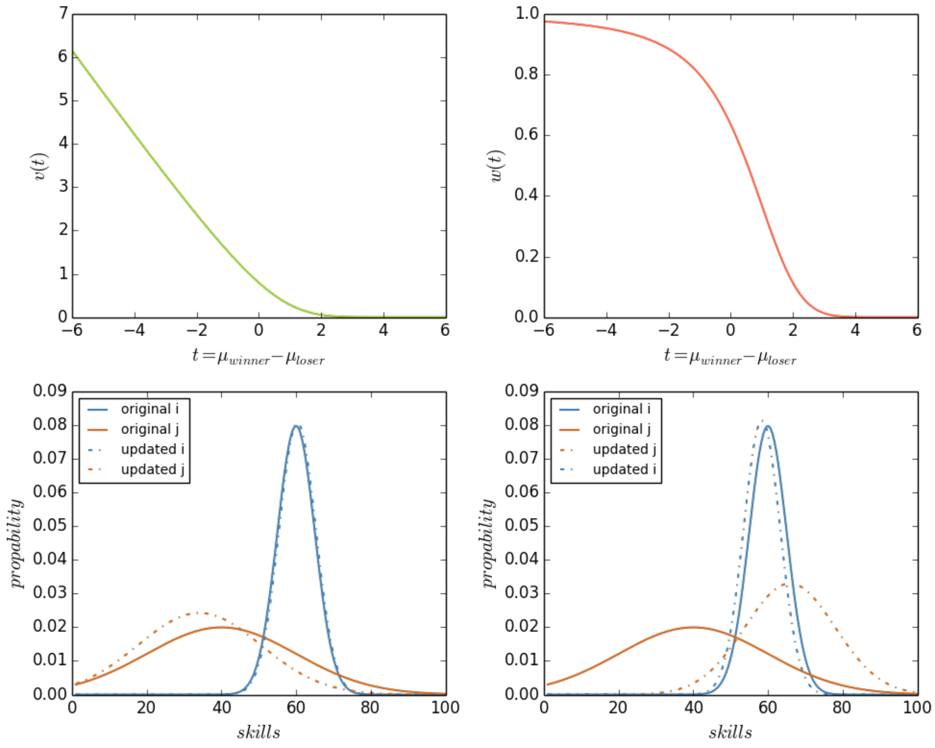

S1 Fig. The demonstration of the TrueSkill system. The two plots in the upper row show how updates increase with surprise (smaller t) and the two plots in the lower row compare the updating between compare the resulting scores between expected (left) and unexpected (right) outcomes.

removing the questions without an accepted answer, there are near 4 million questions and 11 million answers in the dataset.

For each question, we collect all answers to it and define four types of game players: asker, question, accepted answerer, non-accepted answerers. We extract the following four kinds of outcome (we use “>” between winner and loser) for each question:

question > asker	(I)
accepted answerer > question	(II)
accepted answerer > asker	(III)
accepted answerer > non-accepted answerer 1<br>accepted answerer > non-accepted answerer 2<br>...<br>accepted answerer > non-accepted answerer k	(IV)

S1 Table.The four types of outcomes defined in asking and answering activities.

During the updating process, the parameters are set as $\mu = 25$, $\sigma = 25/3$, and $\beta = \sigma/2$ as in [2]. After getting the updating results we calculate $\mu - 3\sigma + 10$ as the final score (we add 10 points for each player to make sure all scores are positive, note that this does not change the ranking of players). The resulting scores quantify the difficulties of questions as well as the skill levels of users, therefore allow us to investigate individual answering strategies, e.g., to compare the kill level of an user against the average difficulty of the questions he chose to answer.

Figure S2 in S1 File shows the distribution of TrueSkill scores of 0.7 million users and 4 million questions (left) as well as the correlation between the TrueSkill scores of users and their reputation score (right). Reputation points `http://stackoverflow.com/help/whats-reputation` are the scores users earn/lose by various activities, such as answering questions, giving comments, etc., thus can be used as an indicator of the skill levels of users to validate the TrueSkill scores. We find that the TrueSkill scores and the reputation of users are positively correlated (Pearson correlation coefficient 0.29, p-value < 0.001, regression coefficient 0.14), supporting the usage of TrueSkill as a rating method.

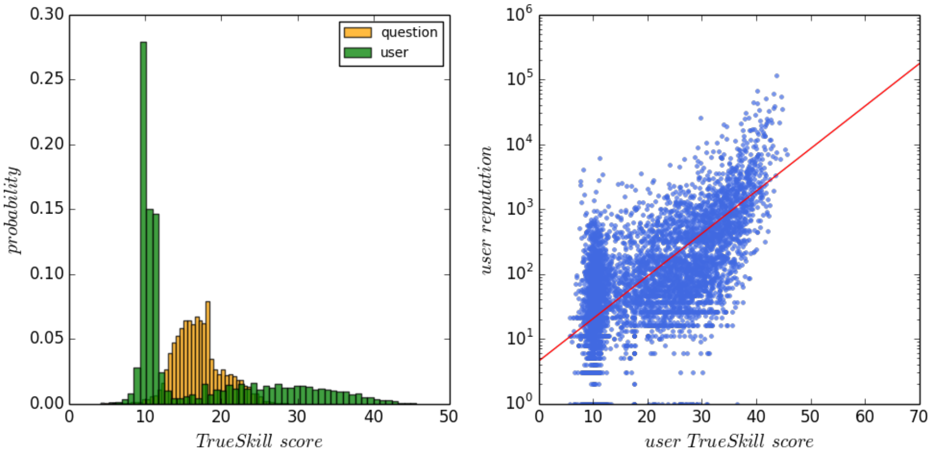

S2 Fig. The TrueSkill scores of questions and users in StackOverflow. The left figure shows the distribution of TrueSkill scores of 0.7 million users and 4 million questions. The right figure shows the correlation between the TrueSkill scores of users and their reputation points (we only show 10,000 data points sampled from the population). We find that the TrueSkill scores and the reputation points of users are positively correlated (Pearson correlation coefficient 0.29, p-value < 0.001, regression coefficient 0.14), supporting the usage of TrueSkill as a rating method.

The dynamics of attention networks

Degree distribution

During the process of network growth, we assume that a new node caring m links chooses a low-degree node with probability a and a high-degree node with probability $1-a$. Therefore, the following number of new links are obtained by the group of k-degree nodes:

$$(a\frac{\frac{1}{k}}{\sum\frac{1}{k}}+(1-a)\frac{k}{\sum k})np_k m\approx(a\frac{2m^2}{k}+(1-a)\frac{k}{2})p_k, \tag{9}$$

We can derive that

$$\frac{p_k}{p_{k-1}}=\frac{\frac{2m^2a}{k-1}+\frac{(1-a)(k-1)}{2}}{1+\frac{2m^2a}{k}+\frac{(1-a)k}{2}} \tag{10}$$

and

$$p_m=\frac{2}{3ma+m+2} \tag{11}$$

When k is very large, $2m^2a/(k-1)$ approximates 0, so we can write Eq. 11 as

$$\frac{p_k}{p_{k-1}}\approx\frac{\frac{(1-a)(k-1)}{2}}{1+\frac{(1-a)k}{2}}=\frac{k-1}{k+\frac{2}{1-a}} \tag{12}$$

Unfolding p_k/p_m gives us that

$$\frac{p_k}{p_m}=\frac{p_{m+1}}{p_m}\frac{p_{m+2}}{p_{m+1}}...\frac{p_{k-1}}{p_{k-2}}\frac{p_k}{p_{k-1}}=\frac{m}{m+1+\frac{2}{1-a}}\frac{m+1}{m+2+\frac{2}{1-a}}...\frac{k-2}{k-1+\frac{2}{1-a}}\frac{k-1}{k+\frac{2}{1-a}}=m(m+1)...\frac{1}{k-1+\frac{2}{1-a}}\frac{1}{k+\frac{2}{1-a}} \tag{13}$$

in which there are h items that contain k. h satisfies

$$k+\frac{2}{1-a}-h=k-1 \tag{14}$$

This is because the numerator of the last item is $k-1$, and k can only be positive integers, so after going back for h items from the end of the equation, $k-h+2/(1-\alpha)$ eventually equals $k-1$. Then the entire list will cancel out, only remaining the first h items whose numerators contain m and the last item whose denominator contains k. So the power law distribution has an exponent approximating $-h$. From Eq. 14 we derive that

$$h=\frac{3-a}{1-a} \tag{15}$$

So the distribution can be written as

$$p_k\approx\frac{2m^{\frac{3-a}{1-a}}}{3ma+m+2}k^{-\frac{3-a}{1-a}} \tag{16}$$

We know that the power-law distribution

$$p(k)\sim k^{-\alpha} \tag{17}$$

can also be expressed in rank-order format (Zipf's law) as

$$k \sim N^{\beta} r^{-\beta} \tag{18}$$

in which r is the decreasing order of k. And the normalized coefficient has to be N^{β} because when $k = 1$, $r = r_{max} = N$. So we can also derived that

$$k_{max} \sim N^{\beta} 1^{-\beta} = N^{\beta} \tag{19}$$

We can derive the relationships between the scaling exponents in Eq 17, Eq 18, and Eq 16 as

$$\beta = \frac{1}{\alpha - 1} = \frac{1 - a}{2} \tag{20}$$

Scaling properties

The number of average links scales to the total number of nodes

We can calculate the expected degree of a linked existed node as

$$E(k) = \sum_{k=1}^{k_{max}} p(k)k = \sum_{k=1}^{k_{max}} (a\frac{\frac{1}{k}}{\sum \frac{1}{k}} + (1-a)\frac{k}{\sum k})k = a\sum_{k=1}^{k_{max}} \frac{1}{\sum \frac{1}{k}} + (1-a)\sum_{k=1}^{k_{max}} \frac{k^2}{\sum k} \tag{21}$$

If we assume that $\sum \frac{1}{k}$ approximates lc, in which l is the number of different degrees and $0 < c < 1$ is the average of $\frac{1}{k}$, Eq. 21 becomes

$$E(k) = \frac{la}{lc} + (1-a)\sum_{k=1}^{k_{max}} \frac{k^2}{\sum k} = \frac{a}{c} + (1-a)\sum_{k=1}^{k_{max}} \frac{k^2}{\sum k} \tag{22}$$

Substituting Eq 19 and Eq 20 into Eq 22 we have

$$E(k) = \frac{a}{c} + (1-a)\frac{\sum_{k=1}^{k_{max}} k^2}{\sum_{k=1}^{k_{max}} k} = \frac{a}{c} + (1-a)\frac{\sum_{r=1}^{N} N^{2\beta} r^{-2\beta}}{\sum_{r=1}^{N} N^{\beta} r^{-\beta}} = \frac{a}{c} + (1-a)N^{\beta}\frac{\sum_{r=1}^{N} r^{-2\beta}}{\sum_{r=1}^{N} r^{-\beta}} \sim N^{\beta} = N^{\frac{1-a}{2}} \tag{23}$$

As the expected degree of the network is $N^{\frac{1-a}{2}}$, if we assume that each new node has an limited "attention quota" approximating constant C and the linking cost to an existed node is proportional to its degree and also moderated by network effect N^h, then the expected number of links obtained by a new node is

$$\Delta m = \frac{C}{N^{\frac{1-a}{2}}} N^h \sim N^{\delta} = N^{\frac{a-1}{2}+h}. \tag{24}$$

The number of new nodes scales to the total number of nodes

In the main text we suggest that the new number of nodes is $E(k)N^{g+1}$, considering Eq. 23 we have

$$\Delta N \sim E(k)N^{g+1} \sim N^{\eta} = N^{\frac{3-a}{2}+g} \tag{25}$$

The number of new nodes scales to the number of new nodes

Putting together Eq. 24 and Eq. 25 we have

$$\Delta M = \Delta m \Delta N \sim \Delta N^{\frac{\delta}{\eta}+1} = \Delta N^{\frac{2(h+g+1)}{3-a+2g}} \tag{26}$$

The following figures show the construction of networks (Figure S3 in S1 File) and the validation of the analytical predictions on degree distribution by data analysis (Figure S4 in S1 File).

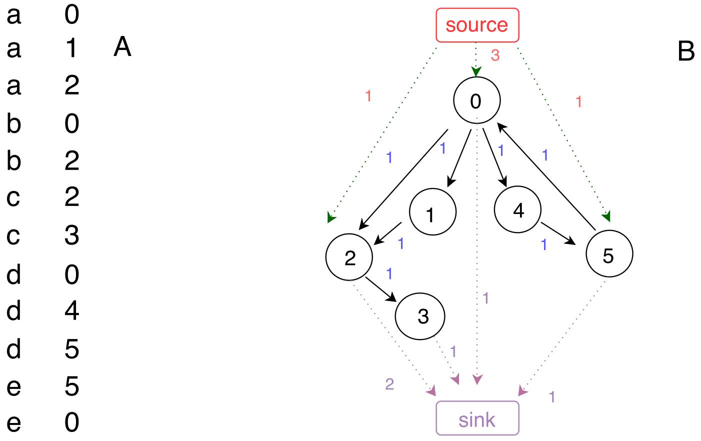

S3 Fig. An example dataset of the StackExchange log file in a day and the corresponding attention network. In (A) the left column shows the anonymized, sorted IDs of users and the right column shows the IDs of the answered questions. In (B) the nodes are questions and the weighted, directed edges are user's successive answering activities between questions. The green arrows show the attention flow "absorbed" from "source" and the purple arrows show the attention flow "dissipated" to "sink". In particular, the network in (B) is constructed as follows. For each record in the dataset, say, [a, 0], if the next record has the same user ID, e.g., [a, 1], we add an edge from node 0 to node 1; otherwise, we create an edge from node 0 to the artificially added node "sink". After all records are converted into clickstreams, we add a "source" node to balance the network such that in-flow (weighted in-degree) equals out-flow (weighted out-degree) over all nodes except "source" and "sink" [6].

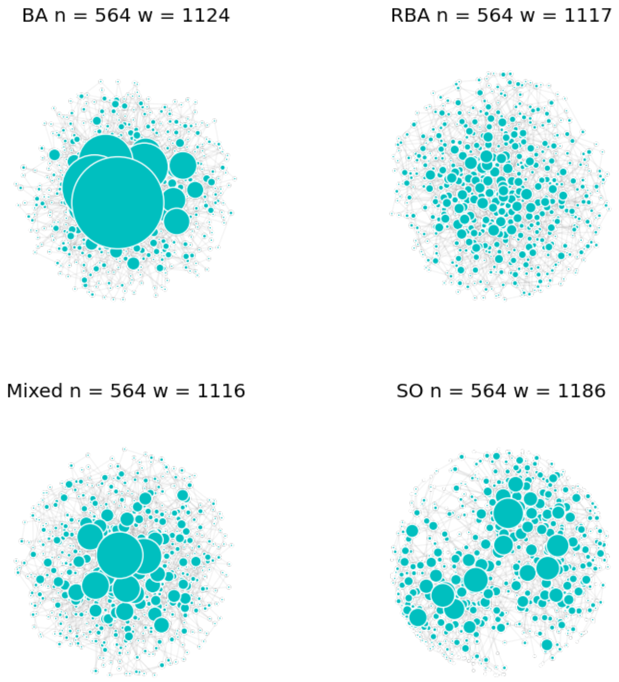

S4 Fig. The simulated networks of three mechanisms and the StackOverflow network. The above figure compares three simulated networks generated by different mechanisms against the empirical network of StackOverflow. The SO attraction network is constructed from the individual question answering records in the first 8 days since the launch of the website. We control the size of the simulated networks as well as the empirical network so that they are more comparable: there are about 500 nodes (questions) and 1000 links (answers) in each of the networks. Please note the differences between the networks in degree distribution (the size of nodes is proportional to the square of degree).

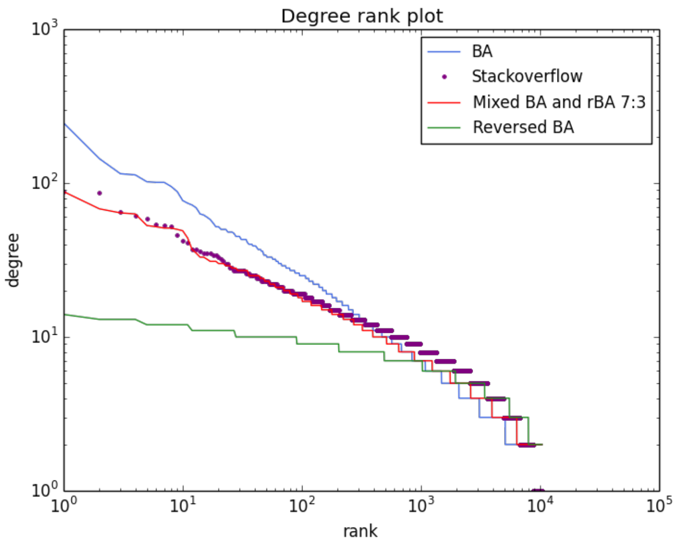

S5 Fig. The degree distributions of simulated networks and that of the StackOverflow network. We show a mixing model in which $a = 0.3$ successfully replicates the distribution of answers across 10381 questions (the cumulative data of SO in 50 days), outperforming the BA model (in which $a = 0$) and the revered BA model (in which $a = 1$). Please note that this value of a is manually tuned to fit a subset of the entire SO dataset, therefore it is not equal to the value of a fitted from the entire dataset.

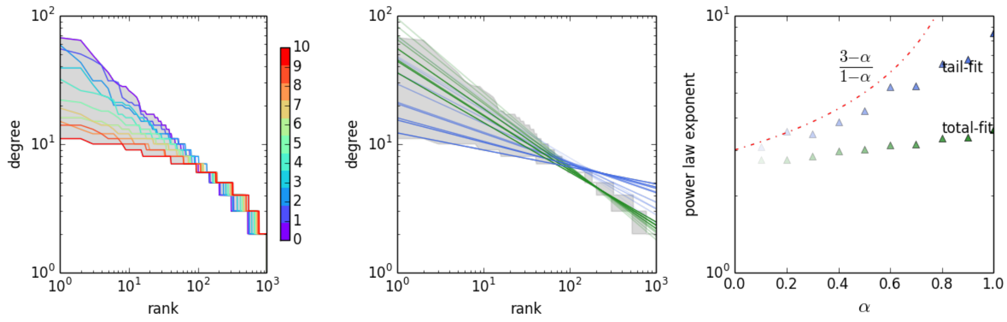

S6 Fig. The degree distributions of mixing models of different parameters. The above figure shows (1) the rank-order plot of degree distributions in simulated attention networks when the mixing parameter $0 < a < 1$ (or, $0 < 10a < 10$); (2) the OLS fitting of the zipf exponent in the log-log axes by total data (blue lines) and tail data (the largest 100 values, green lines). The tail fitting gives the higher explained variance as well as the larger values of a, note that according to our analysis, degree distribution only approximates power-law in the tail. (3) The values of power law exponent vs. the values of a in two kinds of fittings in simulated networks and the theoretical relationship between them (red line). Note that we fitted the data using rank-order plot (Zipf-like), so we need to convert the fitted parameter β into $1 + \frac{1}{\beta}$ in order to obtain the power-law exponent. It is observed that the theoretical curve fits the data better when a is small. This makes sense because when a approaches 1 the power-law like degree distribution degenerates into an exponential distribution.

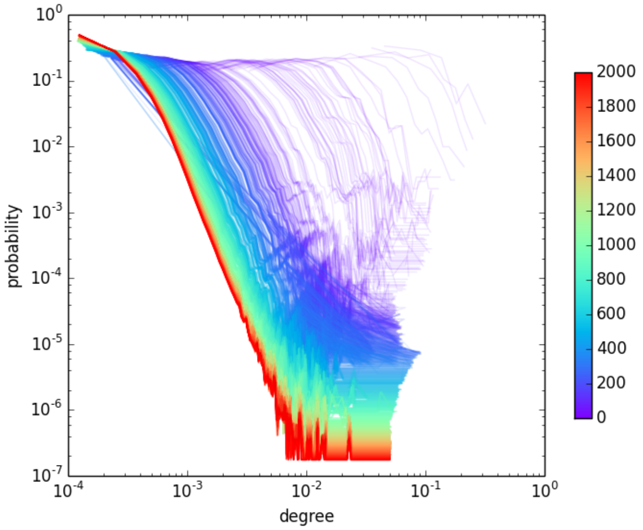

S7 Fig. The evolution of the degree distribution of the StackOverflow network over time. Both axes are shown in log scale. The color bar shows time in days. From this figure we find that the degree distribution converges to a distribution with a power-law tail gradually.

References

1. Elo AE (1978) *The rating of chessplayers, past and present* (Batsford London) Vol. 3.
2. Herbrich R, Minka T, Graepel T (2006) *Trueskill: A Bayesian skill rating system* pp 569–576.
3. Liu J, Song YI, Lin CY (2011) *Competition-based user expertise score estimation* (ACM), pp 425–434.
4. Liu J, Wang Q, Lin CY, Hon HW (2013) *Question Difficulty Estimation in Community Question Answering Services.* pp 85–90.
5. Yang J, Adamic LA, Ackerman MS (2008) *Competing to Share Expertise: The Taskcn Knowledge Sharing Community.*
6. Higashi M (1986) Extended input-output flow analysis of ecosystems. *Ecological Modelling* 32:137–147.
