# Supplementary material for: Correction: The Role of Diverse Strategies in Sustainable Knowledge Production
Source: PLoS One. 2016 Dec 16;11(12):e0168934. doi: 10.1371/journal.pone.0168934 (PMC5161473; doi:10.1371/journal.pone.0168934)
Supplement: S2 File — (PDF) [file pone.0168934.s002.pdf]

PLOS ONE

RESEARCH ARTICLE

# The Role of Diverse Strategies in Sustainable Knowledge Production

**Lingfei Wu**[1]*, **Jacopo A. Baggio**[1,2], **Marco A. Janssen**[1,3]

**1** Center for Behavior, Institutions and the Environment, Arizona State University, Tempe, AZ 85281, United States of America, **2** Department of Environment and Society, Utah State University, 84322, Logan, UT, United States of America, **3** School of Sustainability, Arizona State University, Tempe, AZ 85281, United States of America

* wlf850927@gmail.com

CrossMark
click for updates

OPEN ACCESS

**Citation:** Wu L, Baggio JA, Janssen MA (2016) The Role of Diverse Strategies in Sustainable Knowledge Production. PLoS ONE 11(3): e0149151. doi:10.1371/journal.pone.0149151

**Editor:** Matjaz Perc, University of Maribor, SLOVENIA

**Received:** November 19, 2015

**Accepted:** January 27, 2016

**Published:** March 2, 2016

**Copyright:** © 2016 Wu et al. This is an open access article distributed under the terms of the Creative Commons Attribution License, which permits unrestricted use, distribution, and reproduction in any medium, provided the original author and source are credited.

**Data Availability Statement:** The data set used in the current study is a freely accessible, anonymized dump of all user-contributed content on the Stack Exchange network provided by Stack Exchange (https://archive.org/details/stackexchange).

**Funding:** The authors acknowledge the financial support for this work from the National Science Foundation, grant number 1210856. The funders had no role in study design, data collection and analysis, decision to publish, or preparation of the manuscript.

**Competing Interests:** The authors have declared that no competing interests exist.

## Abstract

Online communities are becoming increasingly important as platforms for large-scale human cooperation. These communities allow users seeking and sharing professional skills to solve problems collaboratively. To investigate how users cooperate to complete a large number of knowledge-producing tasks, we analyze Stack Exchange, one of the largest question and answer systems in the world. We construct attention networks to model the growth of 110 communities in the Stack Exchange system and quantify individual answering strategies using the linking dynamics on attention networks. We identify two answering strategies. Strategy A aims at performing maintenance by doing simple tasks, whereas strategy B aims at investing time in doing challenging tasks. Both strategies are important: empirical evidence shows that strategy A decreases the median waiting time for answers and strategy B increases the acceptance rate of answers. In investigating the strategic persistence of users, we find that users tends to stick on the same strategy over time in a community, but switch from one strategy to the other across communities. This finding reveals the different sets of knowledge and skills between users. A balance between the population of users taking A and B strategies that approximates 2:1, is found to be optimal to the sustainable growth of communities.

## Introduction

Humans are unique in their ability to create public goods in non-repeated situations with non-kin. In larger groups cooperation is more difficult due to the higher temptation to free ride on the voluntary contributions of others [1]. Nevertheless humans are able to create public goods with thousands and even millions of unrelated individuals. For example, there are an increasing number of online communities where participants put in time and effort to make voluntary contributions such as street maps [2], software [3], encyclopedic information [4], protein folding [5], and language translation [6].

Online communities are natural experiments that give us an opportunity to test possible mechanisms that explain cooperation in large groups. Controlled online experiments show

that if participants can choose group members higher levels of cooperation can be derived [7]. This suggests that assortment is a sufficient condition to derive cooperation in large groups. However, such experiments have a duration of about an hour in which participants are all simultaneously online and are recruited with the promise of monetary payments. Whether this scales up to large groups over longer periods of time is an open question.

We will demonstrate in this paper that assortment is not sufficient to derive high levels of contributions in online collaboration [8]. Our analysis shows that at least two different types of strategies of making voluntary contributions are needed to sustain an online community over a longer period of time. One strategy (type A) aims at performing maintenance by doing simple tasks, while the other strategy (type B) aims investing time in doing challenging tasks. We cannot measure the motivations for those two strategies, but we hypothesize that the first may related to reputation in the broader community, and the second to intrinsic motivations and reputation among peers.

For our empirical analysis we investigate the answering records of nearly three million users over a period of six years from 110 online communities. We find that strategy A are important in decreasing the median waiting time for answers, while strategy B users help increase the acceptance rate of answers. Meanwhile, we find that users tend to use the same strategy over time within a communities, but may take different strategies across communities. This can be explained by the different skill sets between users. For example, an expert in science and engineering may try to answer challenging questions in the astronomy community, but only contributes to easy questions in the poker community (see Fig 1). The comparison of overall size across the studied communities suggests that a ratio approximates 2:1 between the population of type A and B users is preferred for bigger communities.

We propose "attention network" models to study the effect of answering strategies on the growth dynamics of communities. In attention networks nodes are questions and edges are the successive answering activities of users that connect two questions. We developed a theoretical network model in which the co-existence of two answering strategies B and A are represented by the mixture of two linking dynamics, i.e., preferential attachment [9] and its "reversed" process [10]. The mathematical analysis of this model not only supports the existence of a trade-off between the two strategies, but also describes the consequences when the mixing ratio in a community deviates from the optimal value. We predict that a community that has too many type A users lacks high quality answers, thus can not attract new questions continuously. On the contrary, a community of too many type B users will attract more new questions than it can handle. In sum, a balance between these two strategies is necessary for the sustainable growth of communities. At the end of the paper, we select three communities as typical cases for analysis, including "math.stackexchange.com" (which has an optimal ratio), "astronomy.stackexchange.com" (which contains too many type A users), and "electronics.stackexchange.com" (which contains too many type B users).

## Materials and Methods

### Data source

Stack Exchange is a network of question and answer communities covering diverse topics in many different fields. We downloaded its database dump on January, 2014 from https://archive.org/details/stackexchange. This data set is a freely accessible, anonymized dump of all user-contributed content on the Stack Exchange network provided by Stack Exchange, Inc. under cc-by-sa 3.0 license (see https://creativecommons.org/licenses/by-sa/3.0/us/ for the explanation of this license). The downloaded data set contained the log files of 110 communities. The smallest community italian.stackexchange.com was created in November, 2013 and

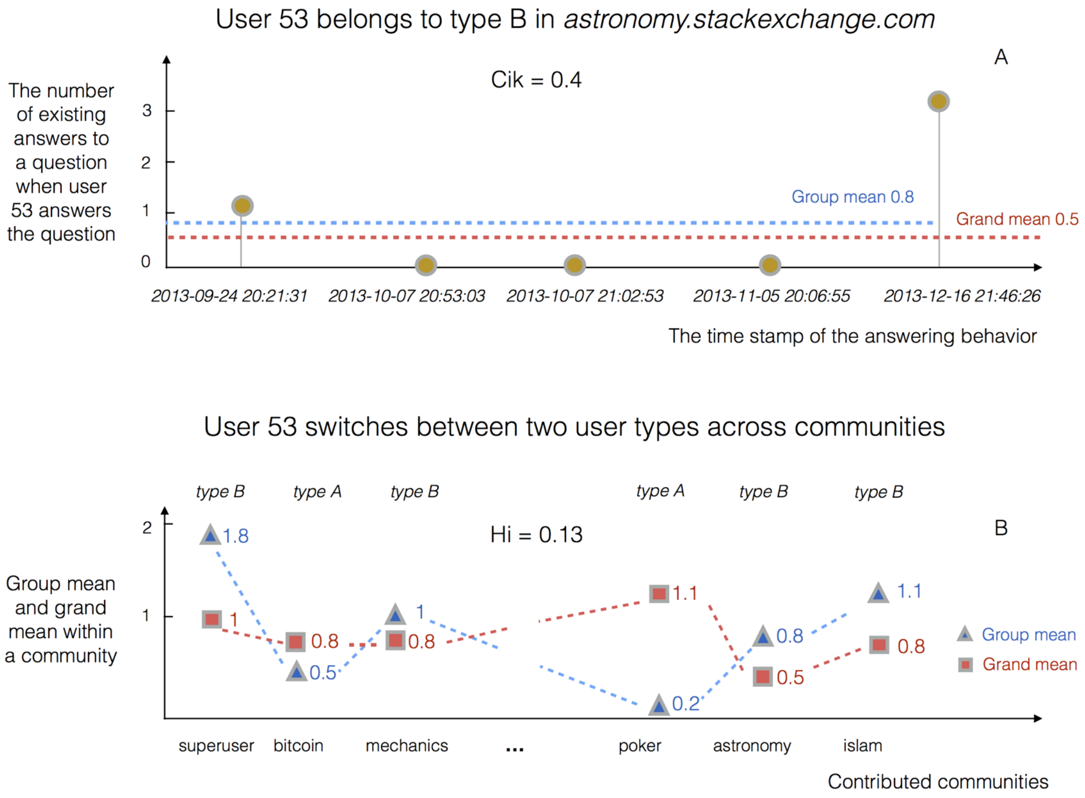


**Fig 1. A demonstration of how to measure the strategic persistence of users.** $C_{ik}$ measures the consistency of the behavior of user *i* within community *k*. As shown in Panel A, user 53 answered $m = 5$ questions in astronomy.stackexchange.com during the time period of observation, and the number of existing answers to these five questions, when they were answered, was $q_{ij} = \{1, 0, 0, 0, 3\}$. Thus we can calculate the group mean of existing answers as $q_i = \frac{1}{m}\sum_{j=1}^{m} q_{ij} = 4/5 = 0.8$. By comparing the group mean $q_i = 0.8$ against the grand mean $E(q_i) = \frac{1}{n}\sum_{i=1}^{N} q_i = 0.5$ of *n* users in the community "astronomy. stackexchange.com", we know that user 53 takes strategy B to answer challenging questions in this community. As for user 53 there are only two out of five answers that satisfy $q_{ij} > E(q_i)$, we calculate the consistency index $C_{ik} = 2/5 = 0.4$. In Panel B, we identify the strategies taken by user 53 in each of the 23 communities he/she contributed to by comparing the group mean $q_i$ (blue triangles) against the grand mean $E(q_i)$ (red squares). It is observed that user 53 takes strategy A in 13 communities and take strategy B in the remaining 10 communities. We identify strategy A as the majority strategy of user 53 and calculate the corresponding fraction 13/23, which is then normalized to obtain $H_i = ((13/23) - 0.5)(1 - 0.5) = 0.13$.

doi:10.1371/journal.pone.0149151.g001

has 374 users, 194 questions, and 387 answers in our data set. The largest site stackoverflow. com (SO) was created in July, 2008 and has 2,728,224 users, 6,474,687 questions and 11,540,788 answers.

Stack Exchange uses a variety of methods to prevent spamming and malicious edits. These methods, including CAPTCHA (Completely Automated Public Turing test to tell Computers and Humans Apart) systems, script detection heuristics, new users limits, collective flagging of spam or offensive flags, auto-removal of items based on flags, and human moderators to handle flagged items, work together to form a human-machine combined system that keeps answers clean and effective [11]. Before analyzing the asking and answering activities of user we cleaned the data such that every user who contributed to attention networks had a unique account in the separated log file containing user profile data. This ensures that the activities under investigation were generated by users who had passed the various anti-spam mechanisms of Stack Exchange.

## Measuring question difficulty and user expertise

We use the number of existing answers as a proxy for the "perceived difficulty" of questions [12] in order to quantify the preference of users for challenging tasks. We firstly count the

number of existing answers $q_{ij}$ to a question $j$ when a user $i$ responds to it. Then we average this number over the $m$ questions answered by user $i$ to derive the average perceived difficulty as $q_i = \frac{1}{m}\sum_{j=1}^{m} q_{ij}$. After that, we use the grand mean of difficulty preferences in a community containing $n$ users, which is $E(q_i) = \frac{1}{n}\sum_{i=1}^{n} q_i$, as the threshold to separate users taking strategy A ($q_i < E(q_i)$) from users taking strategy B ($q_i \geq E(q_i)$) (Fig 2A). To show that these two groups of users are systemically different from each other, we compare them on other three variables, including the average TrueSkill score [13, 14] of answered questions (Fig 2B), the average age of answered questions (Fig 2C), and the acceptance rate of answers (Fig 2D).

The TrueSkill algorithm is a Bayesian ranking algorithm that estimates the skill levels of game players from competition results [13]. Liu et al. first proposed using the TrueSkill algorithm to estimate the difficulty of questions and the skill level of answerers [14]. In our research we use TrueSkill to validate the difference between strategies A and B. In our research we obtain the TrueSkill scores of 912,082 users and 3,771,021 questions in the SO community (please see Figure S1 in S1 File for the details of calculation). As show in Fig 2B, the average TrueSkill scores of questions answered by users taking strategy B are always greater than that of users taking strategy A. (Fig 2B). In our opinion this result validates our division of the two groups. Meanwhile, we find that the TrueSkill scores of users are positively correlated with their reputation points in the log files (Pearson coefficient $\rho = 0.29$, p-value $< 0.001$), justifying the validity of using TrueSkill score as a measure of user skill level.

## Quantifying the strategic persistence of users

We define two indices $C_{ik}$ and $H_i$ to quantify the strategic persistence of users within and across communities, respectively (Fig 1). $C_{ik}$ measures the consistency of the behavior of user $i$ within community $k$. As shown in Fig 1A, for user 53 we calculate the group mean of existing answers as $q_i = \frac{1}{m}\sum_{j=1}^{m} q_{ij} = 4/5 = 0.8$, in which $q_{ij} = \{1, 0, 0, 0, 3\}$ and $m = 5$. By comparing $q_i = 0.8$ against $E(q_i) = \frac{1}{n}\sum_{i=1}^{n} q_i = 0.5$ in the community “astronomy.stackexchange.com”, we can affirm that user 53 tends to adopt strategy B (i.e., answer challenging questions within the community). By analyzing every single answer of user 53 we find that there are only two out of five answers that satisfy $q_{ij} > E(q_i)$. We define $C_{ik} = 2/5 = 0.4$. It is easy to know that $C_{ik}$ may take any values between 0 and 1 and a value close to 1 implies a high level of strategic persistence over time.

We find that user 53 contributes to 23 communities during the time period under investigation. More specifically, he/she takes strategy A in 13 communities and strategy B in the remaining 10 communities. We identify A as the major strategy of user 53 and count the corresponding fraction 13/23. This variable varies from 0.5 (if a user randomly switches between the two strategies across communes) to 1 (if a user sticks to one strategy). We normalize it between 0 and 1 as $H_i = ((13/23) - 0.5)(1 - 0.5) = 0.13$ so it is comparable with the other index $C_{ik}$.

## Constructing attention networks

The answering strategies of individual users can be understood from a network perspective, in which two questions are connected if they are answered sequentially by the same users (see Figure S3 in S1 File for the details). From this perspective, Q&A communities are growing networks with increasing nodes (questions) and links (answers). We call them “attention networks” because they show the transportation of collective attention in solving problems. Attention networks translate answering strategies into linking dynamics; hence provide a quantitative, predictive model for us to explore the collective answering behavior of users.

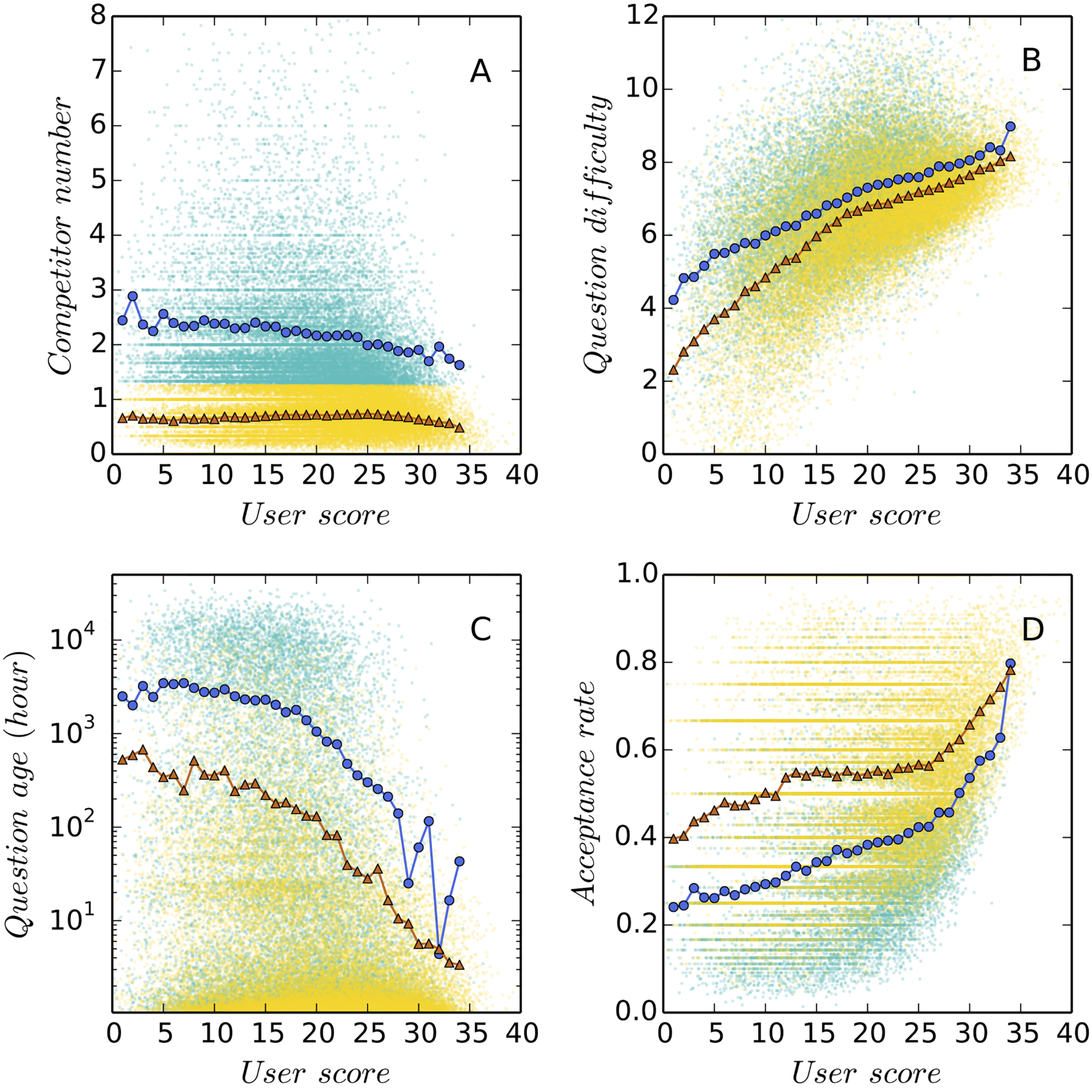


**Fig 2. Two answering strategies in the "stackoverflow.com" (SO) community.** Each data point shown in the background is a user. For each user, we plot four variables against the expertise score calculated using the TrueSkill algorithm [13]. These variables include the average number of existing answers to questions when they are selected by the user under investigation (Panel A), the average difficulty of selected questions (Panel B), the average age of selected questions (Panel C), and the overall acceptance rate of answers (Panel D). We use the mean value of the first variable across all users in the SO community, that is, the number of existing answers to questions (Panel A), as a threshold to separate users into two types, B (higher than threshold) and A (lower than threshold). The proportion of type A users is 0.63 in the SO community. For each of the four variables, the linear-binned data points representing the average values within groups are displayed to facilitate the comparisons between these two answering strategies [15]. It is observed that on average, type A users (triangles) tend to choose newer and easier questions and have higher answer acceptance rates than type B users (circles). See online version for color display.

doi:10.1371/journal.pone.0149151.g002

From empirical data we construct a growing attention network for each of the 110 communities. The network properties we are interested in include the cumulative number of nodes ($N$) and edges ($M$), the daily increments of nodes ($\Delta N$) and edges ($\Delta M$), and the number of links per node ($m = M/N$) and its daily increments ($\Delta m = \Delta M/\Delta N$).

## Results and Discussion

### Two answering strategies and the size of communities

We use the average number of existing answers to questions as a measure of one's tendency to approach challenging tasks and identify two strategies, A and B (see Fig 2 for details). Generally, users taking strategy A prefer easy, new questions and have a higher answer acceptance rate than users taking strategy B, who tend to answer difficult, old questions. To validate our classification on these two types of users we apply the TrueSkill algorithm [13], a Bayesian ranking algorithm that estimates the skill levels of game players from competition results, to calculate the expertise score of users [14]. We find that values of four variables under study, including the average number of existing answers to questions, the average difficulty of questions, the average age of questions, and the acceptance rate of answers, are systematically different between these two groups of users across all expertise levels. Meanwhile, the analysis of variance (ANOVA) on these four variables also supports the assumption that these two groups are significantly different from each other.

Two indices $C_{ik}$ and $H_i$ are proposed to measure the strategic persistence of users within and across communities (see Materials and Methods for details). Both of these two metrics vary from 0 to 1 and the larger value means the higher level of consistency (Fig 1). The idea behind $C_{ik}$ is to identify the strategy user $i$ takes in community $k$ at first, and then count the fraction of answers that support this strategy. To calculate $H_i$ we identify the strategy user $i$ takes in each of the community he/she contributes to, and then select the major strategy used and count the corresponding fraction of communities. We find that $C_i > 0.5$ and $H_i < 0.5$ for an average user in the Stack Exchange system (Fig 3), it means that users tend to stick to one strategy within communities and switch between two strategies across communities. This can be explained by the different skill sets of users. As illustrated in the example presented in Fig 1, user 53, who may be an expert in science and engineering, tries to answer challenging questions in the astronomy community, but has a tendency to answer easy questions in the poker community. We also analyze the relevance between the level of participation and strategic persistence and it is observed that strategic persistence does not change with the number of answers within communities but decreases with the number of communities (Fig 3). This, once again, confirms that users tend to adopt a strategy and be consistent within one community, but tend to switch strategy when answering in different communities.

It is natural to ask, at this stage, whether the mixing ratio of type A and B users has an effect on the overall performance of communities. Two important indicators concerning the performance of Q&A communities, the median waiting time for answers and the overall acceptance rate of answers [11, 17], are considered in the current study. The waiting time is defined as the time elapsed between two events, the posting of a question and the acceptance of the corresponding answer. Median is used as the aggregation scheme, because the distribution of waiting time contains a few extremely large values that may lead to biased results if mean value is used instead [11]. The acceptance rate of answers is defined as the fraction of the questions in a community that find a satisfying (accepted) answer. A good community is expected to have a high acceptance rate of answers and short waiting time[11, 17].

As shown in Fig 4, we calculate the fraction of type A users $a$ ($a \in [0, 1]$) in each of the 110 communities and plot the two above discussed community performance indicators against $a$. It

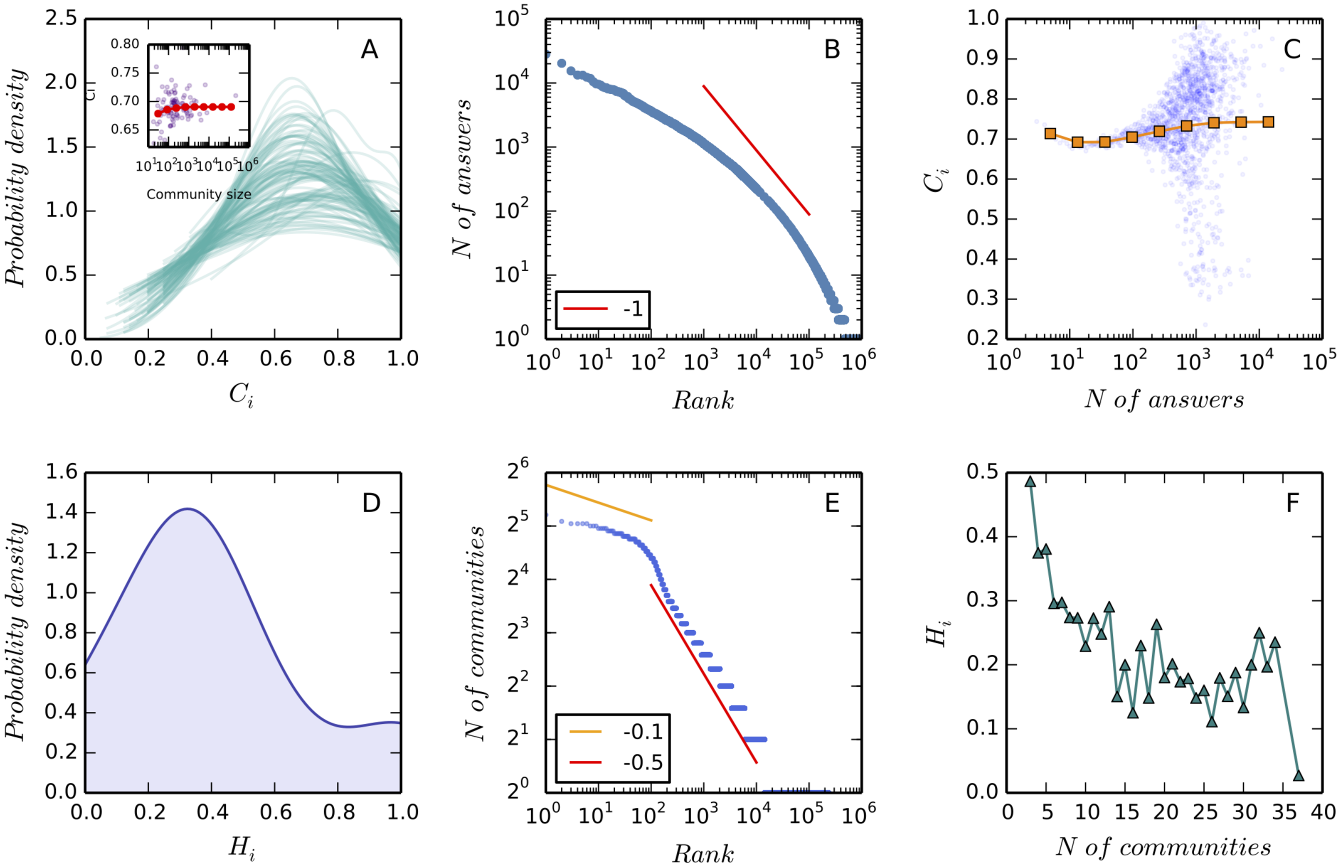


**Fig 3. The strategic persistence of users.** Panel A shows the distribution of $C_i$ for each community $k$, $k$ varies from 1 to 110 (the total number of communities under study). These density curves are obtained by applying the Gaussian kernel density estimation [16] to fit the empirical distributions of $C_i$ (it should be noted that unlike a probability, a probability density function can take on values greater than one). We find that the distributions of $C_i$ are unimodal, with mean values varying from 0.65 to 0.75 across communities. In the inset we plot the mean values of $C_i$ against community size (the total number of contributors in the time period of observation). Each purple point presents a community, and the red circles show the values of $C_i$ averaged within log bins. It is observed that $C_i$ does not change with community size. Panel B gives the distribution of answers contributed by users within communities. This distribution exhibits the "long tail" property but does not follow a simple Pareto distribution (the red line shows a Pareto distribution with exponent −1). The median and mean values of this distribution are 2 and 15.8, respectively. In Panel C we plot $C_i$ against the number of answers for each user (blue dots). The log-binned data (orange squares) is provide to guide the eye. We find that, although the variance of $C_i$ becomes larger within heavy users, the average value does not change dramatically with the increase of contribution. Putting Panel A $\sim$ C together, we conclude that users have a consistent behavior within communities. Panel D shows the distribution of $H_i$ of all users in the Stack Exchange system. This distribution is unimodal and the average value is 0.4. Panel E shows the distribution of the number of contributed communities, which can be modeled as a two-regime power-law distribution [15] (showed by the yellow and red lines). The median and mean values of this distribution are 1 and 1.1, respectively. This means that a majority of users contribute to only one community. Panel F shows that the average value of $H_i$ decreases when users are participating more communities. In Panel D and F we only present the data of the users who contribute to more than three communities as otherwise $H_i$ would trivially equals 1 (if a user only contributes to one community) or switches between 0.5 and 1 (if a user only contributes to two communities). Putting together Panel D $\sim$ E we conclude that users tends to change their types across communities.

doi:10.1371/journal.pone.0149151.g003

is observed that type A users help decrease waiting time and type B users contribute to the increase of answer acceptance rate. Therefore, a balance between these two types of users should be carefully chosen in order to optimize the community performance. In Fig 4C we find that the maximum of community size is achieved by the "stackoverflow.com" (SO) community when $a$ approximates 0.63, i.e., the ratio of type A users to type B users is roughly 2:1. In Fig 4A we plot the number of monthly added questions and answers over time for each community, which are colored by the derivation of the empirical mixing ratios from the optimal value. This figure shows clearly how an oversupply of either type A or type B users leads to an unsustainable growth.

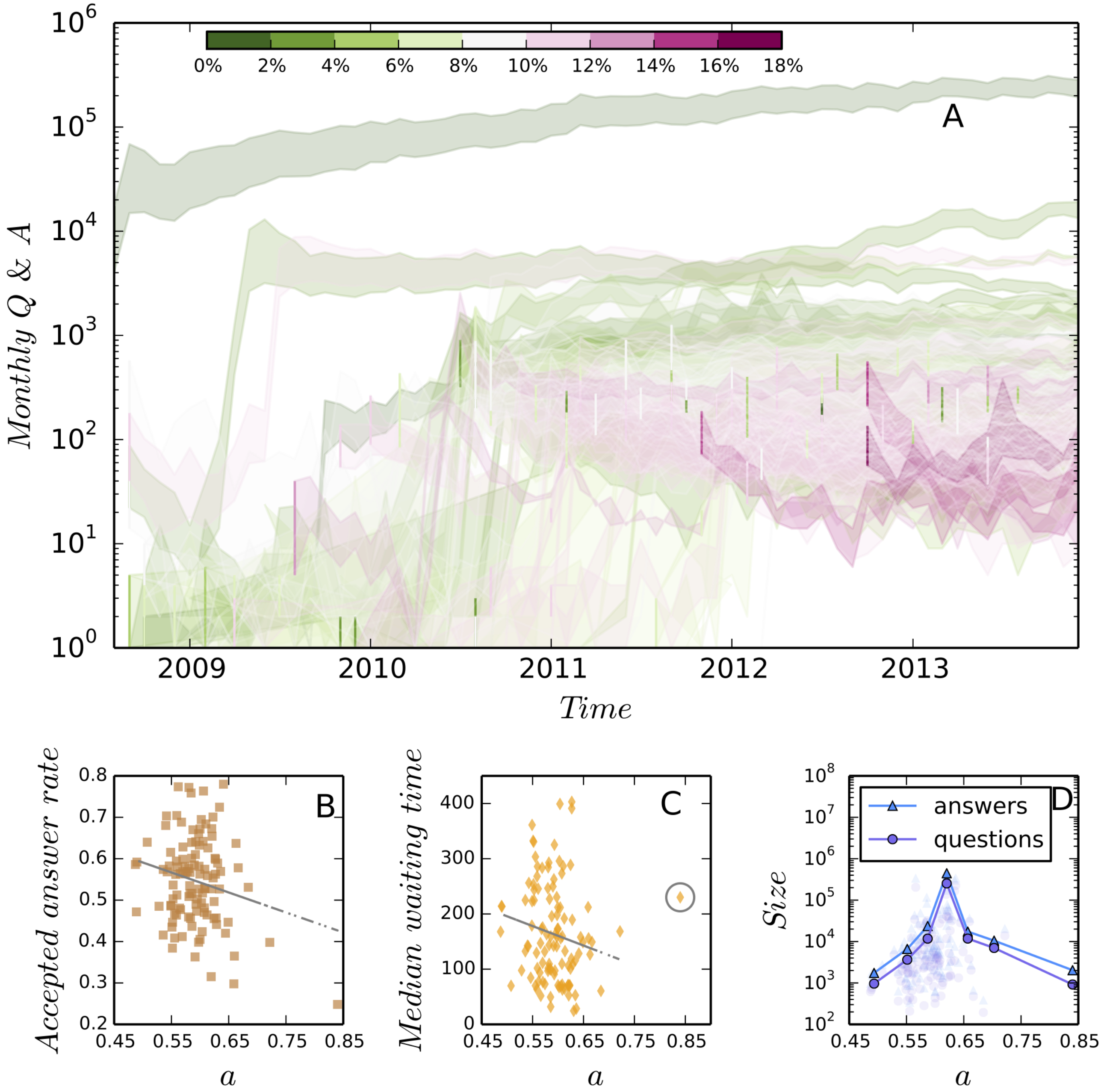


**Fig 4. The effect of the fraction of type A users on the performance of communities.** We find that both of accepted answer rate (B) and median waiting time (C) decrease with the increase of type A users. As a good community is expected to have a high accepted answer rate and a short waiting time of answers [11, 17], our finding suggests that a balance between the two types of users should be carefully chosen in order to optimize the performance. In (B) the regression coefficient is −0.48 and the p-value is 0.029. In (C) after removing the outlier (the data point in the gray circle) the regression coefficient is −352.89 and the p-value is 0.107. Panel (D) suggests that the maximum size of communities is archived when $a \approx 0.63$. Panel (A) shows the monthly increased number of questions and answers of 110 sites, in which the upper bounds of bands show the number of answers and the lower bounds show the number of questions. These bands are plotted in different colors to shown their derivation from the optimal ratio $a = 63\%$. It is observed that the larger deviation from the optimal ratio is related with more unsustainable growth. See online version for color display.

doi:10.1371/journal.pone.0149151.g004

## From answering strategies to linking dynamics

We have shown a co-occurrence between an optimal ratio and the maximum community size. However, why a deviation from this optimal ratio leads to small community size remains

unclear. Therefore, we develop a network model to quantify our assumptions on individual answering strategies in order to analyze the consequences of different ratios.

We use "attention networks" to represent question and answer communities, in which nodes are questions and edges are the sequential answering activities of users. In attention networks, answering strategies based on the number of existing answers to questions can be interpreted as degree-based linking dynamics. As type A users prefer easy questions (low-degree nodes) and type B users favor difficult questions (high-degree nodes), when these two types of users move to a new question from old questions, they bring connections to the new node from old nodes of very different degrees. Therefore, we can naively assume that strategy A corresponds to "preferential attachment" [9], in which the rich get richer, and strategy B corresponds to the reversed process of "preferential attachment" [10], in which the attractiveness of a node decreases with its degree. The reversed "preferential attachment" process has been observed in systems featured by the strong competition between nodes for limited resources, such as food webs [18], power grids[19], and airport networks [20]. For example, in food webs an outbound edge transport resources from a "prey" node to a "predator" node. If several predators are fed on the same prey, it means that the supplied resources have to be split and shared, thus the attractiveness of the prey node will decrease [18]. We argue that this effect also exist in attention networks, in which questions are competing for the limited attention of users.

We use $f$ and $1 - f$ ($f \in [0, 1]$) to represent the probability of observing type A and B strategies, respectively. Note that $f$ is different from the empirical value of $a$ mentioned in the last section, as $a$ was not the fraction of activities but the fraction of users. Using $f$ as a parameter makes our model simple—so that we do not need to take care of the possible non-linear relationship between $a$ and $f$, but our analysis can still provide insights into real systems, as it is easy to understand that $a$ and $f$ must be positively correlated. This is because $f$ can be viewed as the multiplication between two variables, the fraction of users $a$ and answering frequency distribution $w$. As $w$ is always positive, $f$ changes in the same direction as $a$.

Combining "preferential attachment" and its revered version using the mixing ratio $f$, we quantify the probability $p(k)$ of a new question being connected to a existing similar question of degree $k$ as

$$p(k) = f\frac{\frac{1}{k}}{\sum\frac{1}{k}} + (1-f)\frac{k}{\sum k}. \quad (1)$$

In two extreme cases this model degenerates to the "preferential attachment" model ($f = 0$) and the "reversed preferential attachment" model ($f = 1$), respectively. Using the master equation technique [21] we derive that the tail of the degree distribution will converge to

$$p_k \sim k^{-\alpha} = k^{-\frac{3-f}{1-f}}, \quad (2)$$

in which the power exponent $\alpha$ has a minimum value 3 and always increases with $f$. We find that for a majority of communities the empirical value of $\alpha$ lies in the scope of [3, 5] (Fig 5M), supporting our derivation. As a larger power-law exponent implies higher equality in resource distribution [15], our model suggests that type A strategy equalizes the allocation of attention (edges) among nodes and increases the chance of a new question being answered.

Besides degree distribution, the discussed linking rules also explain several scaling relationships observed in the growth dynamics of attention networks as presented by Fig 5 and mentioned in [22, 23]. Users are more likely to post questions when they search the Web and find that a similar question has obtained many answers but their concerns have not been fully addressed. As a consequence, a new question is more likely to be added to the network if the

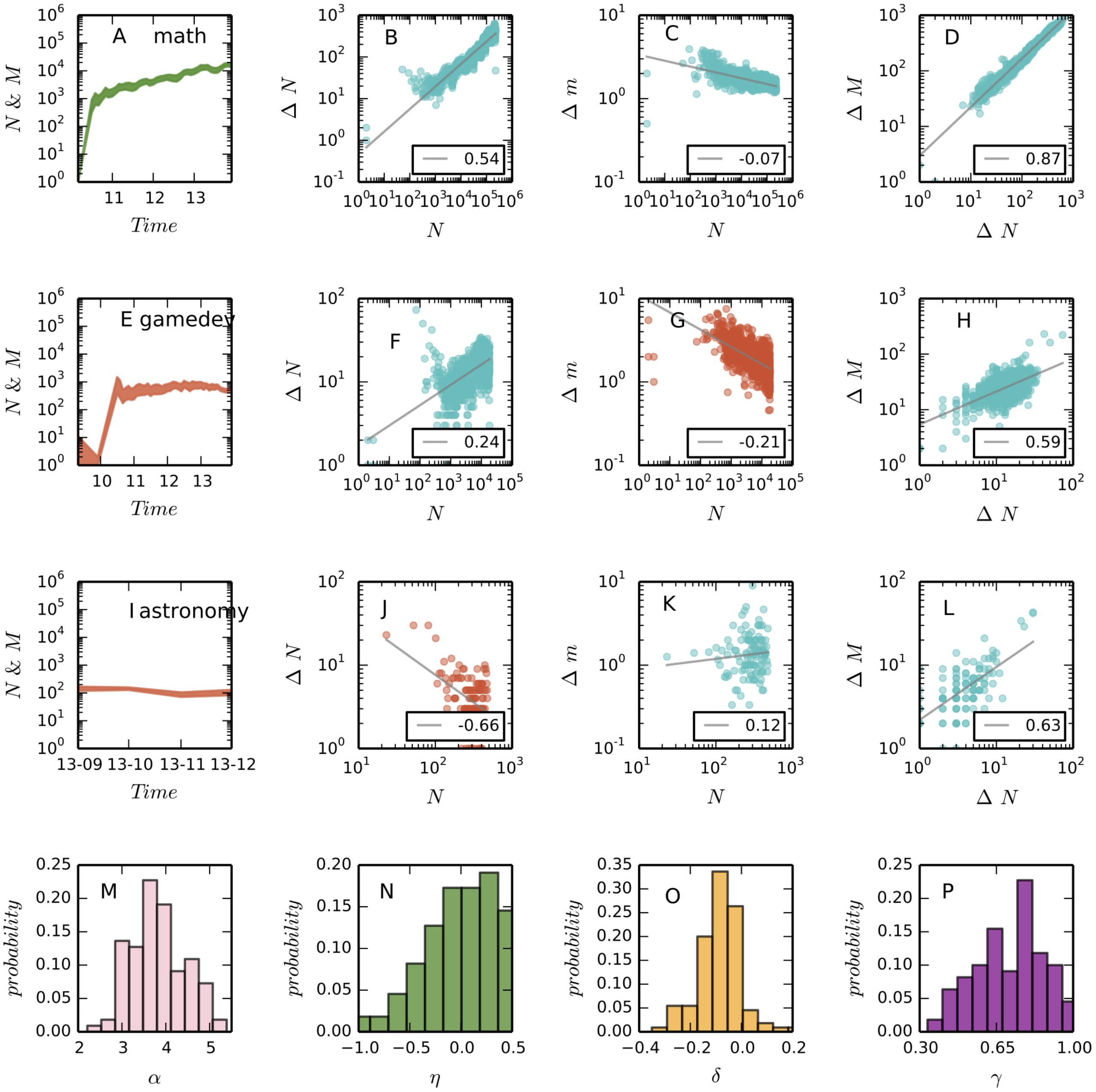


**Fig 5. The scaling properties of communities.** We select "math.stackexchange.com" ($a$ = 0.63, the first row) as the example for sustainable growth and "gamedev.stackexchange.com" ($a$ = 0.62, the second row) and "astronomy.stackexchange.com" ($a$ = 0.67, the third row) as the examples for unsustainable growth. In (A), (E), and (I) we plot the monthly increased number of questions (the lower bounds of the bands) and answers (the upper bounds of the bands) against time. The rest of figures in the first three rows demonstrate the scaling behaviors of communities. In particular, the second column corresponds to Eq 4, the third column corresponds to Eq 5, and the fourth column corresponds to Eq 6. In these figures the OLS regression (gray) lines in logarithmic axes are plotted to demonstrate the estimated scaling exponents. We find that as predicted by our model, in "gamedev.stackexchange.com" new questions increase so fast (F) that they cannot be answered on time (G); while in "astronomy.stackexchange.com" questions are answered quickly (K) but the increase of new questions is very slow (J). The last row gives the distributions of the four scaling parameters across the 110 communities. The mean values of the parameters are 3.7 ($\alpha$), -0.04 ($\eta$), -0.08 ($\delta$), and 0.71 ($\gamma$). See online version for color display.

doi:10.1371/journal.pone.0149151.g005

existing similar questions have more answers. To include this process in our model, we consider node replication as the main driving force underlying network growth and allow high-degree nodes to generate more new nodes. Considering the node-matching probability $p(k)$ given by Eq 1, we can calculate that the expected attractiveness of a single nodes is

$$E(k) = \sum_{k=1}^{k_{max}} kp(k) \sim N^{\frac{1-f}{2}}. \tag{3}$$

Therefore, the expected number of new nodes generated by an existing node is $E(k)N^g$, in which we use $N^g$ to model the effect of network size. By summing $E(k)N^g$ over all nodes in the network we obtain the total number of new nodes as $\Delta N = E(k)N^{g+1}$. Substituting this condition into Eq 3 we derive the scaling relationship between the number of new and old nodes

$$\Delta N \sim N^{\eta} = N^{\frac{3-f}{2}+g}. \tag{4}$$

Note that if an old node generates many new nodes, then there will be a stronger competition between these new nodes for edges. As a result, the cost of linking to an existing node is proportional to its degree [10]. Meanwhile, it is reasonable to assume that new questions cannot obtain an infinite number of answers but have a limited "quota" that approximates constant $C$. Putting these two conditions together, we derive the expected number of links obtained by a new node as $\Delta m = CN^h/E(k)$, in which $N^h$ is the effect of network size. Using the conclusion of Eq 3 we have

$$\Delta m \sim N^{\delta} = N^{\frac{f-1}{2}+h}. \tag{5}$$

From Eqs 4 and 5 we can derive the scaling relationship between the number of new edges and new nodes:

$$\Delta M = \Delta m \Delta N \sim \Delta N^{\gamma} = \Delta N^{\frac{\delta}{\eta}+1} = \Delta N^{\frac{2(h+g+1)}{3-f+2g}} \tag{6}$$

To summarize, the above analysis explains why a balance between strategies A and B is crucial to the growth of communities. On one hand, Eq 5 suggests that a community should have more type A users to maintain the number of answers per question (larger $\delta$); on the other hand, Eq 4 suggests that a community should have more type B users to attract new questions (larger $\eta$). As a balance, Eq 6 predicts that an optimal fraction of type A users, $f = 3 + 2g$, is preferred in order to maximize the value of $\gamma$, i.e., to match the growth of answers with the growth of questions. As $f$ and $a$ are positively correlated, the derived optimimal value of $f$ implies that there is also an optimal value of $a$, which is consistent with our empirical observation.

## Examples of successful and less successful communities

We select three communities to compare the consequences of different mixing ratios between type A and B users, including a community for math questions (math.stackexchange.com, or MATH in short), a community for questions about astronomy (astronomy.stackexchange.com, or ASTR), and a community for questions about game development (gamedev.stackexchange.com, or GAME). As show by Fig 5 (the first column), ASTR and GAME are not as successful as MATH in maintaining a Sustained growth, and this is explained by our model.

The fraction of type A users in MATH is approximately 0.63, which is equal to the optimal value. In contrast, ASTR has more type A users ($a$ = 0.67). According to our model, questions in ASTR will be addressed efficiently, but there will be a slow growth of new questions. This prediction is supported by Fig 5k, which shows that the average number of answers to new

questions is increasing, and Fig 5J, which shows that the increase of new questions slows down as time goes on. In contrast, GAME has a few more type B users ($a = 0.62$) than the optimum. According to our model, in this community new questions will increase fast such that they can not be answered promptly. The fast increase of new questions is evidenced in Fig 5F, and the decrease of answers per question is observed in Fig 5G. It is interesting to note that ASTR is one of the oldest knowledge domains and GAME is a new, fast developing field. Thus, it seems that neither simply being fashion or classic guarantees prosperity, instead, the sustained growth of a community comes from a balance between contributions of diverse users.

## Conclusions

We look at online communities as natural experiments for collective action problems. Our results imply that assortment is not sufficient to derive high levels of contributions in massive collaboration. Instead, strategic diversity seems to be the key for sustainable online communities. In the Stack Exchange datasets, a mixing ratio of 2: 1 between two types of users is found to maximize the size of communities. Type A users have a tendency to answer easy, new questions and type B users prefer to answer difficult, old questions. We propose an attention network model to quantify the answering strategies of users and to explain the existence of an optimal mixing ratio between the strategies. Our conclusion is that type A users contribute to the number of answers and type B users contribute to the quality of answers, thus both of them is crucial to the development of communities.

Our work generalizes the models of Barabasi et al. [9] and Sevim et al. [10] to study large-scale cooperation in online communities. The present analysis on attention networks can also be applied to model a variety of other online collective behaviors such thread browsing [24], photo tagging [25–27], and news sharing [28]. The current study also leaves some limitations behind, which point out the directions of future work. For example, to obtain a simple math model we polarize the rich behaviors of users into two extreme strategies. Meanwhile, in the network model we naively assume that the mixing ratio between the two strategies (linking rules) is a constant over time. In future studies we may consider a ratio that changes with time.

## Supporting Information

**S1 File.**
(PDF)

## Author Contributions

Conceived and designed the experiments: LW MAJ. Performed the experiments: LW. Analyzed the data: LW. Contributed reagents/materials/analysis tools: LW. Wrote the paper: LW MAJ JB.

## References

1. Olson M, Olson M. The logic of collective action: public goods and the theory of groups. vol. 124. Harvard University Press; 2009.
2. Zook M, Graham M, Shelton T, Gorman S. Volunteered geographic information and crowdsourcing disaster relief: a case study of the Haitian earthquake. World Medical & Health Policy. 2010; 2(2):7–33.
3. Schweik CM, English RC. Internet success: a study of open-source software commons. MIT Press; 2012.
4. Jemielniak D. Common Knowledge?: An Ethnography of Wikipedia. Stanford University Press; 2014.

5. Khatib F, Cooper S, Tyka MD, Xu K, Makedon I, Popović Z, et al. Algorithm discovery by protein folding game players. Proceedings of the National Academy of Sciences. 2011; 108(47):18949–18953. doi: 10.1073/pnas.1115898108
6. von Ahn L. Duolingo: learn a language for free while helping to translate the web. In: Proceedings of the 2013 international conference on Intelligent user interfaces. ACM; 2013. p. 1–2.
7. Rand DG, Arbesman S, Christakis NA. Dynamic social networks promote cooperation in experiments with humans. Proceedings of the National Academy of Sciences. 2011; 108(48):19193–19198. doi: 10.1073/pnas.1108243108
8. Henrich J, Boyd R, Bowles S, Camerer C, Fehr E, Gintis H. Foundations of human sociality: Economic experiments and ethnographic evidence from fifteen small-scale societies. Oxford University Press; 2004.
9. Barabási AL, Albert R. Emergence of scaling in random networks. science. 1999; 286(5439):509–512. doi: 10.1126/science.286.5439.509 PMID: 10521342
10. Sevim V, Rikvold PA. Effects of preference for attachment to low-degree nodes on the degree distributions of a growing directed network and a simple food-web model. Physical Review E. 2006; 73 (5):056115. doi: 10.1103/PhysRevE.73.056115
11. Mamykina L, Manoim B, Mittal M, Hripcsak G, Hartmann B. Design lessons from the fastest q&a site in the west. In: Proceedings of the SIGCHI conference on Human factors in computing systems. ACM; 2011. p. 2857–2866.
12. Hanrahan BV, Convertino G, Nelson L. Modeling problem difficulty and expertise in stackoverflow. In: Proceedings of the ACM 2012 conference on Computer Supported Cooperative Work Companion. ACM; 2012. p. 91–94.
13. Herbrich R, Minka T, Graepel T. Trueskill: A Bayesian skill rating system. In: Advances in Neural Information Processing Systems; 2006. p. 569–576.
14. Liu J, Wang Q, Lin CY, Hon HW. Question Difficulty Estimation in Community Question Answering Services. In: EMNLP; 2013. p. 85–90.
15. Newman ME. Power laws, Pareto distributions and Zipf's law. Contemporary physics. 2005; 46(5):323–351. doi: 10.1080/00107510500052444
16. Bashtannyk DM, Hyndman RJ. Bandwidth selection for kernel conditional density estimation. Computational Statistics & Data Analysis. 2001; 36(3):279–298. doi: 10.1016/S0167-9473(00)00046-3
17. Bosu A, Corley CS, Heaton D, Chatterji D, Carver JC, Kraft NA. Building reputation in stackoverflow: an empirical investigation. In: Proceedings of the 10th Working Conference on Mining Software Repositories. IEEE Press; 2013. p. 89–92.
18. Dunne JA, Williams RJ, Martinez ND. Food-web structure and network theory: the role of connectance and size. Proceedings of the National Academy of Sciences. 2002; 99(20):12917–12922. doi: 10.1073/pnas.192407699
19. Amaral LAN, Scala A, Barthelemy M, Stanley HE. Classes of small-world networks. Proceedings of the National Academy of Sciences. 2000; 97(21):11149–11152. doi: 10.1073/pnas.200327197
20. Guimera R, Amaral LAN. Modeling the world-wide airport network. The European Physical Journal B-Condensed Matter and Complex Systems. 2004; 38(2):381–385. doi: 10.1140/epjb/e2004-00131-0
21. Dorogovtsev SN, Mendes JFF, Samukhin AN. Structure of growing networks with preferential linking. Physical Review Letters. 2000; 85(21):4633. doi: 10.1103/PhysRevLett.85.4633 PMID: 11082614
22. Leskovec J, Kleinberg J, Faloutsos C. Graphs over time: densification laws, shrinking diameters and possible explanations. In: Proceedings of the eleventh ACM SIGKDD international conference on Knowledge discovery in data mining. ACM; 2005. p. 177–187.
23. Wu L, Zhang J. Accelerating growth and size-dependent distribution of human online activities. Physical Review E. 2011; 84(2):026113. doi: 10.1103/PhysRevE.84.026113
24. Wu L, Zhang J. The decentralized flow structure of clickstreams on the web. The European Physical Journal B. 2013; 86(6):1–6. doi: 10.1140/epjb/e2013-40132-2
25. Wu L. The accelerating growth of online tagging systems. The European Physical Journal B-Condensed Matter and Complex Systems. 2011; 83(2):283–287. doi: 10.1140/epjb/e2011-20187-9
26. Cattuto C, Loreto V, Pietronero L. Semiotic dynamics and collaborative tagging. Proceedings of the National Academy of Sciences. 2007; 104(5):1461–1464. doi: 10.1073/pnas.0610487104
27. Wu L, Zhang J, Min Z. The metabolism and growth of web forums. PloS one. 2014; 8(9):e102646. doi: 10.1371/journal.pone.0102646
28. Wu F, Huberman BA. Novelty and collective attention. Proceedings of the National Academy of Sciences. 2007; 104(45):17599–17601. doi: 10.1073/pnas.0704916104
